# A Review of Mid-Infrared and Near-Infrared Imaging: Principles, Concepts and Applications in Plant Tissue Analysis

**DOI:** 10.3390/molecules22010168

**Published:** 2017-01-20

**Authors:** Sevgi Türker-Kaya, Christian W. Huck

**Affiliations:** 1 Department of Biology, Faculty of Arts and Sciences, Kocaeli University, 41380 Kocaeli, Turkey; 2 Institute of Analytical Chemistry and Radiochemistry, CCB-Center for Chemistry and Biomedicine, Leopold-Franzens University, Innrain 80-82, 6020 Innsbruck, Austria; Christian.W.Huck@uibk.ac.at

**Keywords:** plants, mid-infrared, near-infrared, imaging, microspectroscopy

## Abstract

Plant cells, tissues and organs are composed of various biomolecules arranged as structurally diverse units, which represent heterogeneity at microscopic levels. Molecular knowledge about those constituents with their localization in such complexity is very crucial for both basic and applied plant sciences. In this context, infrared imaging techniques have advantages over conventional methods to investigate heterogeneous plant structures in providing quantitative and qualitative analyses with spatial distribution of the components. Thus, particularly, with the use of proper analytical approaches and sampling methods, these technologies offer significant information for the studies on plant classification, physiology, ecology, genetics, pathology and other related disciplines. This review aims to present a general perspective about near-infrared and mid-infrared imaging/microspectroscopy in plant research. It is addressed to compare potentialities of these methodologies with their advantages and limitations. With regard to the organization of the document, the first section will introduce the respective underlying principles followed by instrumentation, sampling techniques, sample preparations, measurement, and an overview of spectral pre-processing and multivariate analysis. The last section will review selected applications in the literature.

## 1. Introduction

Plant structures are composed of primary metabolites: carbohydrates, proteins, lipids, nucleic acids, various secondary metabolites and other compounds [1]. These biomolecules are arranged as diverse structural units, which display heterogeneity at different microscopic levels. Such diversity can be between distinct types of tissues, individual cells, and subcellular structures. Under this complexity, the knowledge about biomolecules as composites of organs, tissues and cells is critical for both basic and applied plant sciences. It can be obtained by a variety of analytical methods with microscopic and separation techniques. However, those measurements can be time-consuming, expensive and destructive since plant biologists routinely dissect and process the samples to extract biochemical compounds. In this context, the ability of components of plants to interact with infrared light makes IR spectroscopy a useful tool for quantitative and qualitative analysis with its advantages such as being rapid, non-destructive, reproducible, easy to use and cost-effective [2,3]. However, conventional IR spectroscopy can only provide one average spectrum without any distribution information of the sample’s chemical composition. Therefore, it is difficult to determine whether the components of interest are arising from the bulk sample or from a local region of one sample. In other words, it does not give information on spatial localization of molecular structures at the tissue, cellular and subcellular level. As a result, when considering the heterogeneity of plant structures, the data obtained via this technology may be limited. In this regard, IR imaging/microspectroscopy, which combines IR spectroscopy with visualization, exhibits great advantages for the comprehensive analysis on the spatial distribution patterns of plant constituents [4,5]. Especially, with the use of proper analytical approaches and sampling methods, the techniques provide direct and non-destructive examination while maintaining native compositions of plant samples without the need of extraction, purification and separation steps. Despite their advantages, they are not single molecule detection methods together with requirement of standardization, rigorous data collection and expertise in the chemometrics analysis of IR spectra.

In addition to IR microspectroscopy, a variety of imaging methodologies including Raman microspectroscopy, visible light imaging, fluorescence imaging, 3D imaging, laser imaging, and tomographic imaging (magnetic resonance tomography (MRT), positron emission tomography (PET) and computed tomography (CT)) are also alternatives to analyze plant structures [3]. Thus, by using such technologies, it is possible to gain further insight into the basic plant research related to chemical and physical properties of tissues and samples (e.g., seeds, fruits, grains, leaves, and whole plants) as well as agricultural and horticultural sciences related to localization and quantification of molecules for determination of plant diseases, plant stress due to various factors (e.g., temperature, water, and nutrients). Many reports have appeared in the literature on this topic [4,6,7,8,9,10,11,12,13,14].

## 2. Principles of Mid-IR and Near-IR Imaging/Microspectroscopy

The IR region in electromagnetic spectrum is divided into three regions. These areas are defined as near infrared (near-IR, 13,500–4000 cm^−1^, 780–2500 nm), mid-infrared (mid-IR, 4000–400 cm^−1^; 2500–25,000 nm) and far-infrared (far-IR, 400–10 cm^−1^; 25,000–1,000,000 nm) [15,16]. The wavenumber (cm^−1^) value is converted to wavelength (nm) by the following equation;

(1) ν¯=1λ

where $\bar{\nu }$ is the wavenumber (cm^−1^) and λ is the wavelength (cm = 10^7^ nm).

IR spectroscopy is based on the analysis of IR light interacting with a molecule, which can be analyzed in three different ways as absorption, emission and reflection. Physical basis and development of IR spectroscopy has been extensively documented in recent studies [3,15,17,18,19,20]. Within the scope of this review, a basic introduction concerning mid-IR and near-IR imaging will be given in the following section, but far-IR is outside the focus of the paper.

IR microspectroscopic analysis is the application of IR spectroscopy to obtain spatially resolved image of irradiated sample on a microscope stage. The underlying principle for acquiring such spectroscopic data is the interaction of a sample with propagating light so that the information can be recorded and interpreted from the targeted area. Since an IR spectrum is collected at each pixel in the image, the method is very suitable for analysis of heterogeneous plant samples due to providing data about both the distribution and chemical composition of the components [21,22].

**Figure 1:**
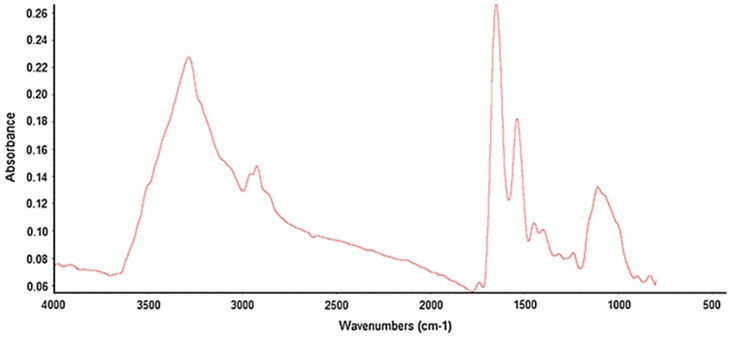
Representative mid-infrared (mid-IR) spectrum of yellow canola (*Brassica napus*) seed. Reproduced with permission from [25], published by Elsevier, 2014.

In mid-IR and near-IR imaging, sample absorbance is recorded at each wavelength. Absorptions in mid-IR spectroscopy correspond to fundamental vibrations of the chemical bonds associated with the atoms of the molecules. Chemical bonds will vibrate more energetically when molecule interacts with IR light, thus causing vibrational and rotational changes in the molecule. However, atoms are constrained by quantum mechanics so that only a few specific energy levels are allowed. The possible rotations are around the axis of symmetry for a given molecule or for either of the two perpendicular axes. If there are only two atoms, the only vibration will be seen as a stretching. When three or more atoms are involved, bonds can also bend [23,24]. Typical mid-IR spectra represent numerous absorbance peaks due to fundamental transitions and is approximately divided into four regions generalized as the X–H stretching region (4000–2500 cm^−1^), the triple-bond region (2500–2000 cm^−1^), the double-bond region (2000–1500 cm^−1^) and the fingerprint region (1500–600 cm^−1^). Finger print region is normally a complex area showing many bands specific to molecular structure of the sample, frequently overlapping each other. Figure 1 shows the absorptions of functional groups in mid-IR region in a plant sample [25].

The peaks in mid-IR spectrum from the vibrations in different functional groups appear in characteristic frequencies of IR (Table 1) [26]. This further facilitates easy band assignment and interpretation with the support of a comprehensive references library of spectra of pure components. In particular, a shifting in band frequency is related to the structural change in molecules under analysis [27]. Thus, plant cell and tissue constituents can be effectively characterized with wide and common use of this technique (qualitative analysis) [26]. In addition, the quantitative information can be obtained from mid-IR spectra since the proportionality of the absorbance (band height or more accurately band area) to the number of functional groups by following the law of Lambert–Beer. This has been previously confirmed by analytical methods for lipid peroxidation products [28] and for some plant constituents [10]. However, it is worth mentioning that despite additional features to conventional IR such as Fourier transform method acquisition that improves the quality of IR spectra, some plant substances in very low concentrations are difficult to be determined by this technique.

**Figure 2:**
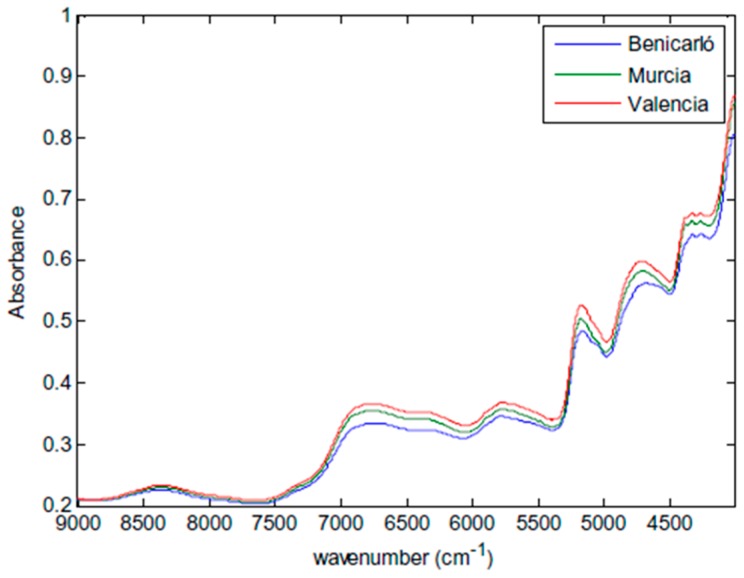
Mean near-spectra of artichoke samples from three different Spanish origins: Castellon (Alcachofa de Benicarló), Valencia and Murcia. Reproduced with permission from [32] published by Elsevier, 2016.

Near-IR region is divided into three regions: Region I (13,500–8500 cm^−1^), Region II (8500–5500 cm^−1^) and Region III (5500–4000 cm^−1^) [29]. The spectral range is narrower than the mid-IR range; therefore, molar absorptivity in near-IR is typically quite small but there is more increased penetration depth of the samples [15,19]. It relies on the vibrations of the molecules described by harmonic and anharmonic motions due to electronic transitions, which categorizes this technique as electronic as well as vibrational spectroscopy. The prominent absorptions in near-IR region are generated by two processes: overtones and combinations of fundamental vibrations of –CH, –NH, –OH (and –SH) functional groups [30]. While the number of possible overtones from a group of absorptions in a molecule is limited to a few, a very large number of combinations are observed [31]. All molecules containing hydrogen atom have a measurable near-IR spectrum, resulting in a larger range of organic materials in plant samples to be suitable for near-IR analysis in comparison to mid-IR [15]. Figure 2 demonstrates the main absorptions in near-IR region in a plant sample. 

The most characteristic near-IR bands of some primary (e.g., carbohydrates, lipids, and proteins) and secondary (e.g., phenolic substances, terpenoids, and alkaloids) metabolites are shown in Table 2 [33].

As demonstrated in Figure 2, peaks are comparatively broader and do overlap. This makes near-IR spectra difficult to interprete during near-IR imaging, making it less useful for qualitative analysis. Nevertheless, there are significant differences among near-IR positions of different functional groups which can often be utilized for quantitative information. However, for such analysis, additional methodologies are required to extract the relevant data while reducing the irrelevant ones [15,34]. In other words, such technique depends on reference methods to separate spectral signatures of sample components and to develop calibration method. The calibration procedure involves acquiring both reference and near-IR data on each sample and deriving a calibration equation by using chemometrics. Once calibration model is developed, a variety of measurement such as moisture content and chemical composition of the sample (e.g., carbohydrates, protein, and lipid) can be obtained from even a single spectrum. As a result, near-IR imaging stands for as a more practical quantitative analytical technique. This contributes to its often more favored usage over mid-IR and other analytical methods employing chemicals, such as gas chromatography (GC) and high performance liquid chromatography (HPLC) [15,24]. 

### 2.1. Instrumentation

**Figure 3:**
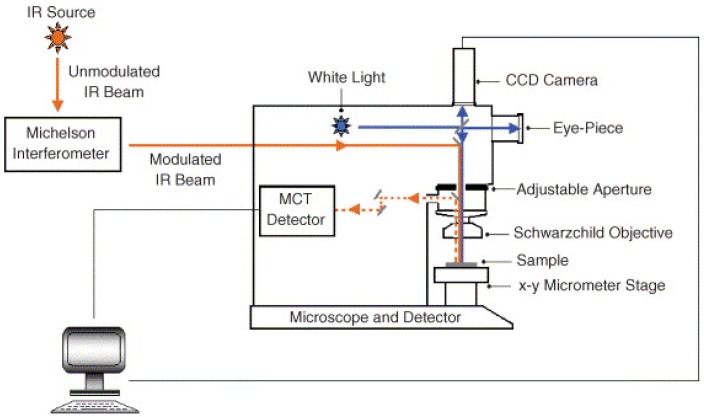
Block diagram of infrared imaging obtained from [44].

Mid-IR and near-IR imaging systems are composed of four main parts as shown in Figure 3.

- *Light source.* A single polychromatic thermal source is heated to 1500–2200 K as light source. Silicon-carbide is used in mid-IR and a tungsten filament in near-IR. Here, it should be mentioned that conventional IR thermal sources only provides a spatial resolution of many tens of micrometers, thus restricting the analysis to tissue level [35,36]. In the case of requiring a resolution of better than 10 micrometers, a synchrotron IR source can be implemented due to emitting 100 to 1000 times brighter IR radiation than conventional sources [37]. Hence, an enhanced spatial resolution and a high signal-to-noise ratio by synchrotron imaging bring greater contrast between adjacent pixels as well as the refinement of having smaller pixel size [13,22].
- *Splitter*. Fourier transforms (FT) interferometers, tunable filters, and diffraction grating spectrometers are three main types used in IR imaging. FT interferometers record information from several wavelengths simultaneously [38] and offer rapid spectral acquisition at high resolution. Filters are used to focus on specific wavelengths and dispense with moving parts in the spectrometer. Tunable filter, as an alternative filter, electronically controls spectral transmission by applying a voltage [39]. The liquid crystal tunable filter is the popular tool for global imaging and mainly used in near-IR hyperspectral imaging. A diffraction grating has a large number of parallel slits separated by a distance comparable to the wavelength of light. Line detectors enable several wavelengths to be acquired at the same time [40]. Narrow slits can reduce the amount of signal reaching the detector whereas large slits might decrease the spectral resolution of the spectrometer. High detector sensitivity and high source intensity in the near-IR range render it suitable for near-IR applications [41].
- *Detector*. Photon detectors are used to record signal after wavelength separation. In near-IR imaging, lead sulfide, indium antimonide, and uncooled indium gallium arsenide are commonly used, while cadmium telluride and mercury are used in mid-IR imaging due to wide spectral sensitivity [38,42].
- *Optics*. Typically, 6×, 15×, and 32× objectives are implemented in mid-IR or near-IR microscope [43].

### 2.2. Sampling Techniques

Mid-IR and near-IR microspectroscopy sampling techniques can be categorized as transmission and reflection. In transmission measurement, the sample is illuminated by source and the detector is placed behind the sample. The sample should be partly transparent. In most cases, this measurement is not possible with thick samples. However, when thin specimens are obtained, the transmission imaging mode provides easy-to-interpret spectra, improves signal-to-noise ratio, decreases the distortion of the spectra, and shows the definite correlation between molecular structures and spectral features. Due to the mentioned reasons, particularly mid-IR imaging has been widely employed in qualitative plant analyses [4]. However, for the transmission measurement, native compositions and physical structures of samples may be altered during preparation [10,44]. In order to overcome this thick plant samples can be investigated by reflection measurement. In such experimentation, the detector is placed on the same side of the sample as the source to record the signal reflected by sample [23]. Most of the time, in reflection tests, original reflection spectra contain the contributions of absorption, reflection, refraction, scattering, and other processes; therefore, some transformation methods such as Kramers–Kronig transformation are required to extract absorption [45]. In mid-IR, diffuse reflection (DR) and the attenuated total reflection (ATR) imaging method are applied. However, the sampling area of ATR imaging is limited by the size of the internal reflection element crystal [45].

### 2.3. Sample Preparation

In mid-IR imaging studies, for most plant samples, thinner sections are usually required to be examined on a calcium or barium fluoride or zinc selenide window. For sample preparation, special care should be given to avoid changes in the chemical composition of the sample. Three sectioning techniques are commonly employed to prepare the samples for imaging. They include Fresh [46], Cryogenic [27,47] and Resin-embedded sectioning techniques [48]. Usually, fresh samples are ideal for minimal manipulation and absence interference from embedding materials. However, here, it should be noted that water absorption bands from high water content of fresh plant samples at 3050–2800 cm^−1^ and 1700–1500 cm^−1^ strongly mask the modes belonging to proteins and lipids. In such cases, samples may be also dehydrated or freeze-dried [49]. Another sample preparation method involves tissue embedding either in a resin or in a paraffin wax, for which tissue fixation is needed. Mostly, resin-embedding of fixed tissue has been used to obtain ultrathin (100 nm to 1 mm) sections as well [48,50]. However, the chemical reagents used for the fixation, embedding, and elution may cause relocation of trace elements and change native chemical compositions and physical structures of the samples [51]. For example, the use of formalin during fixation can oxidize double bonds of unsaturated hydrocarbon chains and cross-link primary and secondary amine groups of proteins. Similarly, paraffin may also cause spectral artifacts in the C–H and C–C vibrations [52]. Even though it is still feasible to use paraffin-embedded plant material after its removal using hexane [53], there is still considerable risk of signal masking due to residual paraffin. Therefore, in the absence of fresh samples, careful sample preparation using a cryogenic sectioning technique is advisable for mid-IR imaging. Cryogenic sectioning can avoid the effects of chemical fixation, dehydration and infiltration of the embedding material into the tissue. It is conducted at low temperatures after embedding the tissue in an organic medium or flash frozen ice with or without fixation. In ice-embedded, flash frozen samples, the vitreous ice formed within the tissue acts as the supporting medium [37]. However, in such a condition, intracellular ice crystal formation can be produced, and thus the sample is damaged.

Comparatively, near-IR imaging having high penetration of the specimen often does not require time-consuming procedures for sample preparation. The sample can be examined either directly or as sections mounted onto window as the same in mid-IR imaging. However, again, overlapping and broad near-IR absorption bands may still necessitate chemometrics to take the relevant spectral data [19,22].

### 2.4. Measurement

The imaging measurement can be performed either in the imaging or mapping mode according to the following strategies.

**Figure 4:**
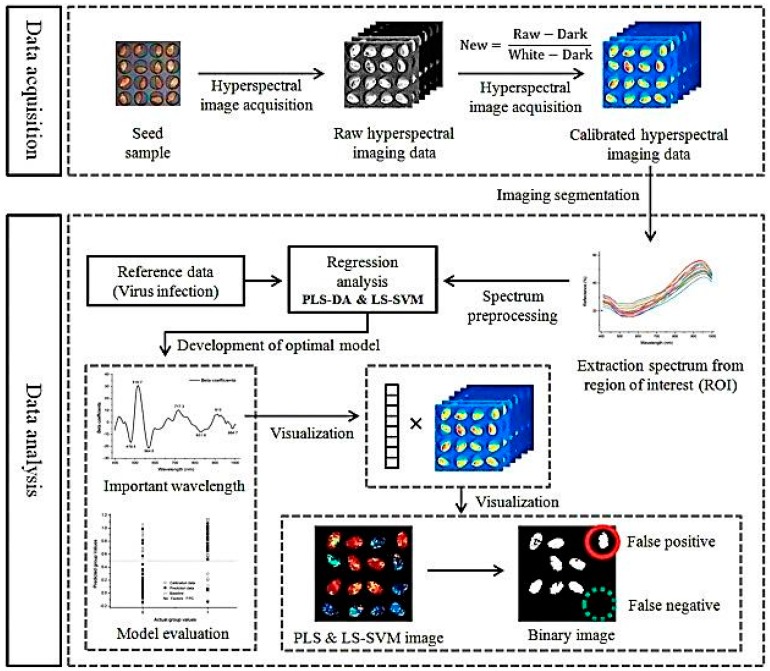
Flow work of hyperspectral imaging for detection of cucumber green mottle mosaic virus-infected watermelon seeds. Reproduced with permission from [57] published by Elsevier, 2016.

- *Point mapping*. A regular grid of spatial positions on the sample surface is defined and a spectrum is measured at one position; and as the sample moves to the next measurement point on the grid, the next spectrum is recorded, and this continues for all positions in the area defining the image. Thus, different areas of the sample are consecutively analyzed.
- *Line mapping*. Spectra are acquired according to predefined spatial positions and the line is moved right to left and up to down to cover the whole area. Subsequently, a series of spectra along one dimension is obtained.
- *Area mapping*. Depending on the overall mapping size, the sizes of the individually analyzed areas, the spectral resolution and the number of repeated scans, mappings with single element detectors can be time-consuming. With Focal Plane Array (FPA), detectors which enable obtaining a series of spectra collected in two dimensions [54], the required measurement time is reduced. These detectors consist of several thousands of single detector elements which record all spectra at once without the need for moving the sample [55,56].
- *Hyperspectral imaging*. The images are acquired at wavelengths in the near-IR region. For this measurement, a huge amount of data is collected in a hyper spectral cube where the three axes include two spatial axes and one spectral axis. This can be generated in one of the four ways: a point-to-point spectral scan in a spatial grid pattern; FT imaging; a line-by-line spatial scan (i.e., the push-broom method); and wavelength tuning with filters. In this cube, the sample is compartmented into small surface or volume areas (referred to as pixels) each of them representing a full spectrum. These cubes are mostly displayed as a three-dimensional matrix or data cube spanning two spatial dimensions, *x* and *y*. The third dimension *z* corresponds to the individual wavelength/wavenumber (Figure 4) [57]. The main disadvantages of hyperspectral imaging include it being costly. Data collection and analysis requires sensitive detectors and fast computers, respectively, and substantial data storage capacity is required due to the size of the hyperspectral images [15].

### 2.5. Quantum Chemical Methods

The application of electronic structure methods, such as quantum chemical methods, for the calculation of frequencies is of great value for the interpretation of complex experimental spectra. Application of quantum chemical techniques molecular orbital theory (MO), damped harmonic oscillator fitting procedure, generalized second-order vibrational perturbation (GVPT2) and density functional theory (DFT) to real systems represents the essence of computational chemistry. Among such approaches, anharmonic vibrational analysis is commonly used for non-fundamental modes. For example, GVPT2 allows acquiring of wavenumber and intensity parameters for first overtones and binary combination modes. The use of this theoretical calculation on the spectrum gives opportunity for fitting procedure. Thus, the information about the number of modes’ components can be obtained with selecting appropriate non-overlapping modes for further analysis. In addition, DFT data offer interpretation of non-fundamental modes. Even though these mentioned methods are needed to extract relevant information from non-fundamental bands, it should be stated that such modes have potential to affect entire spectral regions including mid-IR, for which fundamental bands dominate. For that reason, such analysis should be also performed for mid-IR region. In addition, it has been reported that DFT analyzing non-fundamental modes provides not only analysis for near-IR spectra and but also accurate reproduction of mid-IR region [58,59,60]. Related with this topic, the reports are appeared in the literature [58,61,62]. It is worth mentioning that it these studies, in which such methods are used, were not directly conducted in cells, tissues and organs. However, when considering overlapping IR spectra of plant samples due to fundamental and non-fundamental modes, the implementation of such calculations to IR spectroscopic data obtained from plants will offer accurate interpretation. Relatively, to the best of our knowledge, there is only one work reported by Krichler and co-workers, 2016 [63]. In that study, GVPT2 approach on DFT level was employed for the calculation of near-IR spectrum of the rosmarinic acid of milled *Rosmarini folium* plants. Regarding this study, it will obviously provide a new perspective for the application of such methods to IR imaging data to obtain more efficient interpretation.

### 2.6. Spectral Pre-Processing and Chemometrics

Typical imaging experiments often require the co-addition of many scans to improve signal-to-noise ratio while maintaining high spatial resolution, which may result in the production of 100 or more spectra. The data must therefore be reduced by several image planes and spectral parameters. For this, the first step is pre-processing. It is performed to enhance information and to reduce irrelevant information such as the scattering effects contained in the spectra. These mathematical pre-treatments cover baseline corrections, normalizations, derivatives and smoothing [64,65]. The next steps are classification and cegression. Classification methods are used to group the samples depending on spectral distinctions and similarities. Principal Component Analysis (PCA) is a popular algorithm pattern recognition and discrimination method. When there is no prior knowledge about the sample, PCA is usually the first method to explore the existing signal types in the imaging data. Pixels of the plant sample can be classified according to the score images. However, the principal components are usually not equal to chemical compounds. Therefore, Multivariate Curve Resolution (MCR) methods [4,64,66] are necessary to estimate the spectra and the contents of the chemical compounds from the imaging data. Regression methods link the spectrum to quantifiable properties of the samples. Widely-known regression analyses are Multiple Linear Regression (MLR), Principal Component Regression (PCR), and Partial Least Squares (PLS) [20]. PLS and PCR often lead to very similar results, and MLR performs better when working with a short range of uncorrelated wavelengths or data points. PLS and PCR can be easily adapted for discrimination and are in fact derived from PCA [66,67]. In addition to these, an MCR method called PHAC (PCA-Hierarchical Cluster Analysis (HCA)-Alternating Least Squares-Correlation Coefficients) can be performed to show the spatial distribution of the multiple compositions in each sampling region [4]. Detailed documentation of chemometrics has been provided in the literature [33,67,68]. As mentioned above, near-IR imaging relies on chemometrics to obtain relevant information from broad peaks. Thus, the selection of the reference method is very important when developing near-IR application. On the other hand, with the use of appropriate regression techniques, relationships between absorption values at specific wavelengths and reference values of the constituent could be properly established [33].

## 3. Selected Applications of Mid-Infrared and Near-Infrared Imaging on Plant Studies

This section provides some reports in which mid-IR and near-IR imaging have been employed to investigate phenotypic properties of plant structures for basic plant sciences and agricultural purposes. In order to present a variety of the subjects on which two techniques were used, different applications for each method are described.

### 3.1. Mid-IR Imaging Applications

In comparison to near-IR imaging, mid-IR imaging was developed earlier with conventional light sources to study chemical constituents within plant cell walls [69] and whole tissues [70].

#### 3.1.1. Identification of Cell Wall Components

The chemical specificity of mid-IR region allows the identification of certain peaks in 1200–950 cm^−^^1^ region related to cell wall components [29]. Taking advantage of this, Fourier Transform Infrared Microspectroscopy (FT-MIR) was utilized to study the alterations in cell wall components in different tissues throughout plant development and growth. For instance, Barron et al. (2005) [71] compared hard and soft wheat (*Triticum aestivum*) endosperm textures, and found the presence of higher amounts of a water-extractable arabinoxylan in the peripheral endosperm of soft grains. In another study, the changes in similar molecules during cell-elongation were monitored to characterize maize (*Zea mays*) coleoptile growth by application of neural networks on mid-IR spectra [1]. Moreover, the differences occurred in the ratio of β-1,3-1,4-glucans/arabinoxylans, and in the arabinoxylan in endosperm grain during the stage of development was reported by Saulnier et al. (2009) [72]. Similarly, an increase in cellulose obtained from peaks at 900 and 1320 cm^−1^ and a decrease in pectins denoted by modes at 1014, 1094, 1152, 1238, and 1741 cm^−1^ were obtained during *Arabidopsis* embryo germination [73]. Likewise, a study of *Luffa cylindrical* multiple pollen tubes formation from a single pollen grain has revealed lower amounts of pectins and accumulation of abnormal cell wall components related to lignin, pectin, cellulose, callose and overall cell wall carbohydrate content [74]. FT-IR microspectroscopy was also used to determine post mortem lignification of tracheary elements of *Zinnia elegans* by monitoring 1510 and 1595 cm^−1^ [75]. Recently, a combination of FT-MIR and an FPA together with chemometric analysis was performed to characterize different secondary xylem cell types (vessels, fibers, and rays) across the annual wood ring of aspen (*Populus tremula*) and to monitor changes in the cell walls. In this study, in fiber cells, lignin predominated in early wood and hemicelluloses/cellulose in late wood during the growing season. Additionally, xylem ray cells were found to contain more aromatic compounds (lignin and monolignols) in early wood and more pectins and/or hemicelluloses in late wood [76]. Furthermore, Dokken and Davis, 2007 [13] employed synchrotron radiation infrared microspectroscopy (SR-IMS) to probe cellulose, lignin, and proteins, in the root tissue of hydroponically grown sunflower and maize plants. Upon the application of PCA, epidermis and xylem tissues of two different plants could be discriminated. The authors revealed that the best successful separation of maize and sunflower tissues was achieved depending on the band at 1635 cm^−1^ attributed to hydrocinnamic acid in (H type) lignin. A similar technique was conducted to identify the acetyl esterification of the cell walls of the black cotton-wood (*Populus trichocarpa*). According to the results, *p*-coumarate accumulated in young leaves and declined in mature leaves, while ferulate and acetate were predominantly found in stems. Over the course of stem development, the amount of ferulate increased, whereas the initial amount of *p*-coumarate diminished [77]. Again, related with cell wall components, ATR FT-IR microspectroscopy was applied in conjunction with multivariate statistical analysis on petal samples of *Petunia hybrida* from wild-type and from two transgenic lines in which the *PhEXPA1* expansin gene expression was down-regulated and up-regulated to determine the role of expansin in the rearrangement of the cell wall polymer network. Within the scope of the study, the changes in cell wall composites absorption bands assigned to pectin were as follows: 1740 cm^−^^1^, 1595 cm^−^^1^, 1440 cm^−^^1^, 1150 cm^−^^1^, 1105 cm^−^^1^ and 975 cm^−^^1^; hemicellulose: 1260 cm^−^^1^, 1230 cm^−^^1^ and 1075 cm^−^^1^; and cellulose: 1025 cm^−^^1^. The data revealed that compared to wildtype there was a decrease in down-regulated samples for pectin and cellulose but in up-regulated samples for hemicellulose [78]. Lastly, the alterations in cell wall components were detected to monitor the degradation of spruce wood by brown-rot fungi (*Gloeophyllum trabeum* or *Poria placenta*) by transmission FT-IR imaging microscopy and multivariate analysis. The findings showed that brown-rot starts to become significant in the outer cell wall regions (middle lamellae, primary cell walls, and the outer layer of the secondary cell wall S1). Most significant during incipient decay was the cleavage of glycosidic bonds, i.e., depolymerization of wood polysaccharides and the degradation of pectic substances. Accordingly, intramolecular hydrogen bonding within cellulose was reduced, while the presence of phenolic groups increased [79].

#### 3.1.2. Protein Structure Analysis

In mid-IR spectra, amide I and amide II modes are attributed to proteins. Since the height of amide I is very sensitive to changes in proteins structures, the changes in secondary structure elements can be predicted [26]. Related with this, FT-IR synchrotron infrared microspectroscopy was used to determine protein structure of leaves of *Lc*-transgenic and non-transgenic alfalfa using Gaussian and Lorentzian methods of multi-component peak modeling. HCA and PCA as well as curve fitting analysis revealed that transgenic alfalfa contained a relatively lower percentage of alpha helices and beta-sheets and a higher percentage of turns and random coils. On the other hand, transgenic *Lc*-alfalfa leaves had similar proteins with non-transgenic alfalfa obtained by no difference in cluster and PCA analysis [80]. Similar approach was also done on endosperm in hard wheat breeding by Bonwell and co-workers (2008) [81], on seed proteins in the wild type and T-DNA insertion mutants of *Arabidopsis thaliana* cv. by Withana-Gamage et al. (2013) [40] and on feed barley varieties by Yu et al. (2006) [82]. Identical system was also utilized to quantify protein damage caused by frost (Xin et al., 2013) [27].

#### 3.1.3. Tissue and Taxa Differentiation

Huck-Pezzei et al. (2012) [10] used similar tool with clustering techniques (*k*-means clustering, fuzzy *c*means clustering and HCA) to differentiate morphological and molecular patters of different tissues of St. John’s wort (*Hypericum perforatum*) by monitoring lipids (1740 cm^−1^), phospholipids (1240 cm^−1^), proteins (1630 cm^−1^, 1550 cm^−1^), carbohydrates (between 1185 and 930 cm^−1^), and nucleic acids (1080 cm^−^^1^). Depending on the contents of the components such as epidermis, phloem, protoxylem, sclerenchyma, and xylem tissues were successfully discriminated. Parallel distinction had been previously done on barley tissues such as percarp, seed coat, aleurone, and endosperm by detecting lignin, protein, carbohydrates, cellulosic materials and lipids [83]. The capability of the same technique combined with agglomerative HCA and PCA was tested to distinguish endosperms of barley grain, corn grain and wheat by analyzing 1720–1485 cm^−1^ (protein), 1650–950 cm^−1^ (non-structural CHO starch) and 1185–800 cm^−1^ (total CHOCO vibrations). The findings revealed that effective discrimination of three samples could be achieved based on protein mode but not the other modes [84]. In addition, Dell’Ann and co-workers (2010) [85] distinguished pollens of eleven taxa by using FT-IR transmission microspectroscopy together with unsupervised and supervised multivariate statistical methods depending on the wavenumber range of 4000–850 cm^−1^. In this study, HCA provided reproducibility of the FT-IR spectra of the same taxon. In supervised method, best results were obtained from a *K*-nearest neighbors’ classifier and the leave-one-out cross validation procedure on the dataset composed of single pollen grain spectra (overall accuracy 84%).

### 3.2. Near-IR Imaging Applications

#### 3.2.1. Discrimination of Different Plant Samples

Even though near-IR imaging is widely applied for quantitative analyses with the use of chemometrics, it has also been utilized for discrimination of plant samples. For example, dried fruits of two different species *Illicium verum* (Chinese star anise) and *Illicium anisatum* (Japanese star anise) could be distinguished by short-wave infrared hyperspectral push-broom imaging (920–2514 nm) system and multivariate analysis. In this study, a classification model with four principal components and an R^2^X cum of 0.84 and R^2^Y cum of 0.81 was developed for the species using Partial Least Squares Discriminant Analysis (PLS-DA). The model was used to predict the identity of *I. anisatum* (98.42%) and *I. verum* (97.85%) [86]. The same technique in conjunction with PLS-DA, soft independent modeling of class analogy (SIMCA), *K*-nearest neighbor algorithm (KNN) and support vector machine (SVM), and random forest (RF) was also applied to distinguish rice seed samples based on 1039 nm and 1612 nm spectral range. For classification, PLS-DA and KNN models represented over 80% accuracy, and SIMCA, SVM and RF models produced 100% accuracy in both the calibration sample sets [87]. Similarly, different maize kernels (e.g., hard, intermediate or soft) from inbred lines were properly classified using Matrix near-IR camera (960–1662 nm) and the Sisu Chema short wave IR hyperspectral push-broom imaging system (1000–2498 nm) [12]. The authors were obtained with mean square error of prediction 0.18 and 0.29, respectively by PLS-DA. Liu and Giu (2015) [88] employed hyperspectral imaging together with chemometric analysis to identify kiwifruits treated with exogenous plant growth regulator in the range of 865.11–1711.71 nm. PCA, Successive Projections Algorithm (SPA), PLS regression and SVM modeling methods were used to select principal components and characteristic wavelengths. The results indicated that average correct identification rates of all models were higher than 98.9% and 96.7% for the calibration set and validation set, respectively. The best model was found to be PLS-SPA whose average accuracy rate reached 100% for the calibration set and 98.4% for the validation set.

#### 3.2.2. Measurement of Biomolecule Related Parameters

One of the studies measured content of biomolecules in plant samples by using near-IR imaging was performed by Yu et al. (2014) [8] to determine the spatial distribution of total nitrogen in leaves, stems, and roots of pepper plants. Within the scope of the study, Total Nitrogen Contents (TNCs) were measured using Dumas Combustion (DC) method. RF algorithm was implemented to select the wavelengths (992, 756, 749, 918, 909, 921, 758, and 912 nm) that represented the best prediction for different tissues of TNCs. PLS model was used to build the quantitative relationship between the spectral reflectance and TNCs-DC of samples based on full spectra and selected wavelengths. The RF-PLS model of the whole-plant with results of RP 50.876% and RMSEP 50.426% was considered as the optimal model for the TNCs-HSI prediction in pepper plants. TNCs-HSI of all pixels in samples was calculated by applying the optimal PLSR model. In another report, the same technique was also conducted for quantitative identification and distinction of aflatoxin-infected maize kernels from clean ones [89]. The authors adopted the masking method to reduce the noise after pixel-level calibration, and then conducted inverse PCA and secondary PCA to enhance the signal-to-noise ratio. Upon interactive analysis, two PCs were found to indicate the spectral characteristics of healthy and infected maize kernels, and the wavelengths of 1729 and 2344 nm were also identified to indicate aflatoxin exclusively. The n-dimensional visualization method based on PC3 to PC7 was adapted to separate the aflatoxin-infected and clean kernels. The result was compared with chemical analysis of Aflatest, and the verification accuracy of pixel level reached about 100%. In addition, ElMasry et al., 2007 [14] tested a Vis/near-IR (400–1000 nm) hyperspectral imaging system to determine the moisture content, total soluble solids, and acidity in strawberries. Furthermore, Schmilovitch et al. (2014) [90] studied three cultivars of bell pepper (“Ever Green”, “No. 117” and “Celica”). The quality parameters including total soluble solids, total chlorophyll, carotenoid and ascorbic acid content were determined during maturation by hyperspectral imaging in 550–850 nm regions. Comparisons were made between the PLS regression analysis of the reflectance spectra (R), and the first derivative spectra (D1R), log(1/R), D1(log(1/R)) and D2(log(1/R)). High correlations were obtained by the established models with the coefficients of 0.95, 0.95, 0.97, and 0.72 for total soluble solids, total chlorophyll, carotenoid and ascorbic acid content, respectively. Moreover, the quantification and localization of glucosinolates in florets of a single broccoli species were examined by hyperspectral imaging in the regions of 950–1650 nm [91]. By using the same technique, nitrogen deficiency was identified by chlorophyll concentration distribution map of cucumber. PCA was performed to reduce the dimension along the wavelength axis. MLR was used to build calibration models relating the spectra and chlorophyll concentration which was further confirmed by HPLC. Chlorophyll concentration was reasonably well-predicted with a high correlation (*R* = 0.8712). Distribution of chlorophyll concentrations on the nitrogen deficient and control cucumber leaves were obtained. Thus, the results concluded that hyperspectral imaging exhibits considerable promise for non-destructive diagnostics of nitrogen deficiency in cucumber plant [92]. Similar approach combined with chemometric methods was performed by Liu et al. (2015) [93] to measure lycopene and phenolic compounds content in intact tomatoes. PLS, least squares-support vector machines (LS-SVM) and back propagation neural network were applied to develop quantitative models. Compared with PLS and LS-SVM, back propagation neural network (BPNN) model considerably improved the performance with coefficient of determination in prediction (R^2^P) = 0.938 and 0.965, residual predictive deviation (RPD) = 4.590 and 9.335 for lycopene and total phenolics content prediction, respectively.

#### 3.2.3. Detection of Bruises and Tissue Damages

Hyperspectral imaging has been also utilized to identify bruises and damages on different agricultural products. For instance, bruise damages on “Shingo” pears were detected by application of a classification algorithm based on *F*-value. The optimal waveband ratio for the discrimination of bruises was obtained as 1074 nm and 1016 nm: R1074/R1016 with the accuracy of 92% [94]. The same system was also performed to detect bruised areas on strawberries [95] and on wheat kernels [96]. Additionally, bitter pit lesions on apples [97] and fungal infection by *Penicillium digitatum* in citrus fruit [98] were efficiently detected in combination with PLS model.

#### 3.2.4. Analysis of Firmness of Fruits

Lu et al. (2005) [99] had earlier used a push-broom multispectral imaging system (400–1000 nm) to determine the scattering profiles of soft and firm “red haven” peaches. Experiments showed that soft fruit possesses broader scattering profile than firm ones and their difference was most pronounced at 680 nm. Likewise, firmness of apples was identified by a Multispectral Imaging (MSI) system over the wavelengths at 685, 850, 904 and 980 nm. In the scope of the study, to validate the MSI system, intra-lipid solutions of known concentration were used. The best MSI results of R = 0.87 and root mean square error of cross validation (RMSECV) = 7.17 were obtained when the Lorentzian model parameters derived at each wavelength were combined using MLR [100].

#### 3.2.5. Endosperm Texture Determination

To evaluate endosperm texture of hard, intermediate, or soft whole yellow maize kernels a linescan (push-broom) instrument in the wavelength from 1000 to 2498 nm was tested [11]. After multivariate image cleaning PCA model was performed following multiplicative scatter correction (MSC) and mean-centering. Different clusters representing vitreous and floury endosperm (different types of endosperm present in varying ratios in hard and soft kernels) and a third type of endosperm were obtained in the score plot of the second and fourth principal components. Chemical interpretation revealed that starch density and the protein matrix could be monitored for differentiation of endosperm textures. The vitreous and floury endosperm clusters were used to make PLS-DA model, using four components, with a coefficient of determination (*R*^2^) for the *y* data (kernel hardness category) for the training set of over 85%. 

#### 3.2.6. Assessment of Plant Development

Germination periods of muskmelon seed were predicted by near-IR hyperspectral imaging by push-broom system in the spectral range of 948–2494 nm. The spectra from seeds on Germination Days 3 and 5 and non-germinated seeds were studied for development of PLS-DA. Most effective wavelengths were selected using Variable Important in Projection (VIP), Selectivity Ratio (SR), and Significance Multivariate Correlation (sMC). The PLS-DA model was constructed using individual VIP, SR, or sMC variables. The results demonstrated that the PLS-DA model, which was developed with the selected optimal variables from the different methods, provided comparable results for the calibration set; however, the PLS-DA-SR method afforded the highest classification accuracy (94.6%) for a validation set used to determine the viability and vigor of muskmelon seeds [101]. More examples for application of hyperspectral imaging in crop and plant analysis are available in a review provided by Cozzolino and Roberts, 2016 [18].

### 3.3. Combined Studies

For imaging measurement, the transmission imaging mode presents easy-to-interpret spectra with high signal-to-noise ratio. However, the native chemical compositions and physical structures of plant samples may be altered during microtome process as stated above. To overcome this direct measurement of thick plant samples by the combination of near-IR and mid-IR imaging can be proposed. Chen et al. recently took advantage of near-IR transmission and ATR microspectroscopic imaging to characterize non-microtomed *Ginkgo biloba* leaves. In order to interpret ATR imaging spectra, PCA, HCA, alternating least squares (ALS), and PLS were used. Near-IR transmission microspectroscopic imaging was applied to explore the primary chemical structures of whole leaf samples and the C–H and O–H bonds in secretory cavities, veins, and mesophylls were found to be different due to overlapped modes. Instead of this, mid-IR images indicated that secretory cavities and mesophylls contained more resin-like compounds and proteins, whereas veins contained more cellulose and calcium oxalate. In addition, within the scope of the study, the surface chemical composition leaf samples were also studied by ATR microspectroscopic imaging. The spectra and distribution images of cuticle, protein, cellulose, ginkgolic acids, and calcium oxalate on the adaxial surface were obtained using the PHAP procedure, which is the combination of PCA, HCA, ALS and PLS. As a result, chemical compositions of secretory cavities, veins, and mesophylls were analyzed in detail, and the distributions of some compounds on the surface layers of *Ginkgo biloba* leaves were revealed [9]. 

In another experiment, the chemical morphology of the Areca nut section was directly characterized by the reflection near-IR imaging and the attenuated total reflectance mid-infrared (ATR-MIR) imaging methods. According to NIR spectra, there are three types of pixels corresponding to the endosperm, perisperm, and testa, respectively. In addition to these data, the endosperm and perisperm contain a lot of carbohydrates and lipids as well as a few protein particles, while the testa mainly consists of tannins obtained by ATR imaging results. Thus, in the content of the study, first, the reflection near-IR imaging explored the sample to find out typical regions in small sizes. Then, ATR-MIR imaging method measured the typical regions and revealed the molecular structures and spatial distributions of compounds of interest [4].

## 4. Summary and Future Outlook

Mid-IR and near-IR imaging are non-destructive versatile analytical techniques in plant analysis providing qualitative and quantitative data with spatial distribution patterns of components in heterogeneous cells, tissues and organs. Two technologies have different advantages and constraints. The mid-IR has the practical limitation of thin specimen and difficult sample preparation. In contrast, near-IR data are more readily obtained with minimal sample preparation while offering deeper sample penetration. The appearance of mid-IR absorptions from vibrations of different functional groups in characteristic frequencies of IR facilitates easy band assignment and interpretation. Thus, plant composites can be qualitatively analyzed with wide and common use of this technique. Comparatively, near-IR interpretation usually requires statistical multivariate data treatment due to broader overlapping modes. However, once calibration method is developed, quantitative information about chemical composition of plants can be obtained, which is an ignored advantage of this technology over other conventional methods. Thus, taking the advantages of these methods several characteristics and properties of plant structures have been analyzed. 

Even though the benefits of the techniques over other analytical methods are sensitivity, analytical speed, and minimal sample preparation and easy operation, they still require development. Particularly, the use of multivariate data analysis methods and sampling techniques to interpret the data generated may bring some restrictions their use. As future perspectives, the development of the technologies in terms of methodologies and instrumentation will facilitate their applicability for a wide range of plant sciences. The developed theory covers several factors such as higher performing instrumentation, algorithms and software for signal processing. In particular, even though they are well-established as laboratory systems, there is still need for miniaturization. This will enable on-site and in-field analysis, which offers great opportunity to study the plant in their natural environments. Additionally, advances in instrumentation like improvement in resolution will increase their sensitivity, and thus efficacy of investigation. Further developments in chemometrics and quantum chemical methods will obviously contribute to easy spectral analysis. 

In sum, the integration of mid-IR and near-IR imaging with basic and applied plant research can overcome bottleneck issues due to receiving the full benefit of the available molecular data. In this way, reliable, automatic, multifunctional, and high-throughput plant phenotyping platforms may be developed more, and thus, plant scientists may gain new insights into all the aspects of plants.

## Figures and Tables

**Table 1:** General band assignments of mid-IR spectrum of plants based on the literature.

Frequency (cm^−1^)	Definition of the Spectral Assignments
3500−3200	O-H and N-H stretch: carbohydrates, proteins, alcohols and phenolic compounds
2960−2950	CH_3_ asymmetric stretching: mainly lipid with a little contribution from protein, carbohydrate, and nucleic acid
2930−2920	CH_2_ asymmetric stretch: mainly lipid with a little contribution from protein, carbohydrate, and nucleic acid
2875−2870	CH_3_ symmetric stretch: mainly protein with a little contribution from lipid, carbohydrate, and nucleic acid
2860−2840	CH_2_ symmetric stretch: mainly lipids with a little contribution from protein, carbohydrate, and nucleic acid
1745−1730	Saturated ester C=O stretch: phospholipid, cholesterol ester, hemicellulose, pectin, lignin, suberin/cutin esters
1650−1630	Amide I (C=O stretch): protein, pectin, water associated cellulose or lignin, alkaloids
1630−1620	C=C stretch: phenolic compound
1610−1590	C=O aromatic stretch: lignin, alkaloid
1560−1540	Amide II (C=N and N–H stretch): mainly protein
1515−1505	C=C aromatic stretch: lignin
1460−1455	Amide III (aromatic hydrocarbons): mainly protein
1455−1440	C–H asym bending of CH_2_ and CH_3_: cell wall polysaccharide, lipid and protein
1430−1420	O–H bend: cell wall polysaccaride, alcohol, and carboxylic acid
1380−1370	C–H sym bending of CH_2_ and CH_3_: cell wall polysaccharide, lipid and protein
1375−1365	C–H bend: cellulose and hemicellulose
1250−1240	C=O stretch: pectic substances, lignin, hemicellulose, suberin/cutin esters
1235	Amide IV (C=N and N–H stretching): mainly protein
1235−1230	C–O stretch: lignin, xylan
1205−1200	O–H in plane bend: cellulose
1170−1160	C–O–C asym stretch: cutin
1160−1150	Symmetric bonding of aliphatic CH_2_, OH, or C–O stretch of various groups: cell wall polysaccaride
1145−1140	C–O–C asym stretch: cellulose (β-1.4 glucan)
1110−1105	C–O–C sym stretch: cutin
1105−1100	Antisymmetric in-phase: pectic substance
1085−1075	C–O deformation: secondary alcohol, aliphatic ester
1075−1070	C–O ring stretch: rhamnogalactorunan, b-galactan
1065−1060	C–O stretch: cell wall polysaccarides (glucomannan)
1045−1030	O–H and C–OH stretch: cell wall polysaccarides (arabinan, cellulose)
990−980	C–O stretch: cutin
900−890	C–H deformation: arabinan
895−890	C–O valence vibration: galactan
875−870	C–O stretch: β–d-fructose

**Table 2:** General band assignments of near-IR spectrum of plants based on the literature.

Wavenumber (cm^−1^)	Wavelengths (nm)	Definition of the Spectral Assignments
8403	1190	C–H str. first overtone: carbohydrates
8251	1212	C–H str. second overtone: carbohydrates
7375	1356	2 C–H str. + C–H def.: carbohydrates
7168	1395	2 C–H str. + C–H def.: carbohydrates
6983	1432	N–H str. second overtone: proteins
6748	1482	O–H str. first overtone: carbohydrates
6662	1501	N–H str. first overtone: carbohydrates
6494	1540	O–H str. first overtone (intermol. H-bond): starch
6394	1564	N–H str. first overtone: proteins
6196	1614	C–H str. first overtone: carbohydrates
6053	1652	C–H str. first overtone: carbohydrates
5896	1696	C–H str. first overtone: carbohydrates
5627	1777	C–H str. first overtone: plant fiber composed of cellulose, lignin and other carbohydrates
5507	1816	O–H str. + 2 C–O str.: plant fiber composed of cellulose, lignin and other carbohydrates
5120	1953	C–O str. second overtone: carbohydrates
4878	2050	N–H sym. str. + amide II: proteins
4824	2073	O–H str. + O–H def.: alcohols
4643	2154	Amide I + amide III: proteins
4439	2253	O–H str. + O–H def.: starch
4363	2292	N–H str. + CO str.: proteins

str.: stretching, def.: deformation.
